# Phase field modelling of hopper crystal growth in alloys

**DOI:** 10.1038/s41598-023-38741-2

**Published:** 2023-08-03

**Authors:** P. C. Bollada, P. K. Jimack, A. M. Mullis

**Affiliations:** 1 School of Computing, University of Leeds, Leeds, UK; 2 School of Chemical and Process Engineering, University of Leeds, Leeds, UK

**Keywords:** Materials science, Mathematics and computing

## Abstract

Here we use phase field to model and simulate “hopper” crystals, so named because of their underlying cubic structure but with a hopper-like depression on each of the six faces. Over the past three decades simulations of single phase solidification have successfully explored dendritic structures, in two and three dimensions, formed under high undercooling from a slight perturbation in anisotropy. More recently we see the modelling of faceted structures at near equilibrium, and also, under high undercooling, the formation of dendritic-like structures in two dimensions which retain some faceting in the dendrite arms. A cubic hopper crystal appears to be a hybrid structure, somewhere between a perfect cube and a dendrite, and, to date, has not appeared in the modelling literature. In this paper we describe a model for faceted cubic growth and explore results, necessarily in three dimensions, that include perfect cube, hopper and dendritic. We also touch briefly on one other morphology—octahedral.

## Introduction

The established view in modelling crystal growth is that the morphology of equilibrium crystals are convex Wulff shapes corresponding to a given surface energy^1–5^, so it follows that growth of non-convex crystals must be non-equilibrium phenomena produced under non-equilibrium solidification conditions. Dendrites, for example, are by their very nature non-convex. Moreover, it has also been shown in^6,7^ that even crystals with underlying faceted morphology grow dendritic-like structures under rapid solidification conditions. This might mark the end of the subject if it was not for the variety of 3D structures that seem to be neither dendritic nor convex. For example, in^8^ back-scatter electron images of heterogeneously nucleated Cu6Sn5 in Sn-Cu-Al solders reveals several morphologies that might fall under the better description: *skeletal crystals*. In figure 5 of^8^ the Cu-Al intermetallic forms deep depressions in the faces of a cube, alongside Cu-Sn which forms a long hollow channel. The authors’ description of the depicted images as having *depressions near their facet centre*, hardly fits the description of “dendrite”. So it remains a puzzle as to the origin of morphology that seems to fit neither equilibrium-faceted nor a dendritic description. In^8^ the authors put forward a mechanism for these particular shapes: *most likely due to slower growth in facet centres and faster growth at edges and corners where solute diffusion is more effective at preventing solute build-up. This growth instability then promotes hopper crystals... * . Other possible mechanisms put forward for hopper crystal formation include^9–11^ but the current state of understanding is probably best described in^12^: *The growth of hopper crystals is observed for many substances, but the mechanism of their formation remains ill understood*.

A hopper crystal is one of the more commonly seen skeletal crystals, and is so named because the faces resemble a hopper machine - large at the input and small at the exit.

In general hopper crystals can form in different materials under different physical scenarios: (1) intermetallic alloy solidification; (2) single component materials such as bismuth, quartz (called skeletal or fenster crystals), gold, calcite, and (3) common salt crystal formation from salt solution. However, there are an abundance of materials that are never seen as hopper crystal: all of the solid solution alloys for example. So what singles out the mechanism for hopper growth as opposed to faceted or dendritic growth?

One of the earliest descriptions of hopper growth can be found in^14^, and is that of growth resembling a spirally terraced hill. In^15^ there is an example of the terracing in an optical photomicrograph. SEM images of calcite hopper crystals are reproduced in^16^ revealing both the hollowing and terracing. As imaging methods improved^17^ examined PbS crystals synthesized by hydrothermal reaction and an FESEM image also reveals partly formed hopper crytals with a characteristic break in the edge. Perhaps the most ubiquitous hopper crystals are found in salt crystals:^18^ give an overview SEM image of NaCl spherulites with hollow spherical architecture forming in crate-like groups, and therefore exhibiting very low average density. Salt crystals are also observed in^12^ and^19^. Also^20^ have observed images that correspond closely to the hopper crystals described in^14^, occasionally growing in pairs or blocks. This is also seen in^21^, which also discusses an important application of hopper crystal—catalysis. This is in large part due to a high surface area which raises the possibility of other applications, for example, of lightweight materials, or enhancing the delivery of poorly soluble pharmaceuticals. In the case of hopper shaped salt, the increased surface area is reported to give an enhancement of taste (per unit salt used)- an image of a typical salt hopper crystal can be seen in Fig. 15b of^13^.

Higher conductivity materials Iron-Carbon-Silicon as shown in^13^, Ceasium based alloys in^22^, and Cu6Sn6 observed in^8^ are also possible and raise the question whether the mechanism in metals is the same or similar as the formation of salt crystal from solution. Hopper-like growth need not be restricted to cubic, $<111>$, morphology, and there are observations of related skeletal growth in the SEM observations of^23,24^.

The other ubiquitous hopper crystal found in nature is that of bismuth, a reproduction of which is found in^10^. This is used as an illustration alongside a discussion of the mechanism of formation of different crystal surfaces attributed to the relative latent heat of fusion, $l\equiv L/(RT)$, where *L* is the latent heat of fusion, *T* is the equilibrium melting temperature, and *R* the molar gas constant. When *l* is low dendritic growth building on rough surfaces is preferred; and when high—smooth growth; with hopper growth illustrated by a bismuth hopper crystal—a midway case.

It should be observed that^10^ long predated the introduction of anisotropy into phase field modelling. Indeed, modelling faceted crystal growth is only a relatively recent development^6^, with the ideas, in the context of more general morphologies only becoming clear in^25^. Since then, as far as we are aware, there has been no modelling or simulation of out of equilibrium 3D faceted growth, and particular hopper growth simulation has not been attempted.

Without modelling, the mechanism for formation of hopper crystals cannot be properly understood, with the current explanation being that growth is inhibited on the faces by build up of a condition detrimental to growth, but which leaves edges and vertices unrestricted. Key players in the mechanism are thought to be enthalpy of crystallisation and surface energy, but to date it is not clear whether thermodynamic considerations alone are sufficient to describe this morphology.

The above observations motivate the simulation of solidification under non-equilibrium conditions, where the equilibrium shape is faceted and we seek amongst the resulting morphologies those with a hollow crystal appearance. We investigate whether it is possible to simulate alloy growth into a hopper shape, whilst assuming high (indeed infinite) thermal conductivity by imposing a constant thermal field.

The paper is set out as follows: we first state the phase field model as used, including all free parameters in section “The phase field model”, but without a discussion of the surface energy, which is addressed separately in section “Phase field modelling of faceted anisotropy”. The treatment of anisotropy is an approximation to the method presented in^25^, and meets the computational challenge of approximating a function defined as a maximum of a list of functions. The results are presented in section “Results” and there is supplementary material with appendices, A to E, that elaborate on statements made in the text.

## The phase field model

The non-dimensional phase field model used in this paper (see supplementary Sec. B of the supplementary material for its association with standard dimensional units, e.g. S.I.) is given by the evolution equation for phase, $\phi \in [0,1]$

1 $$\begin{aligned} \frac{1}{M}{\dot{\phi }}= & {} \nabla \cdot \frac{\partial {}}{\partial {\nabla \phi }}\left( \frac{1}{2}A^2\right) - \frac{\Omega '(\phi )}{\delta ^2}-\frac{g'(\phi )(\mu _0-\mu )\Delta c}{\lambda \delta ^2} \end{aligned}$$

where the double well potential, $\Omega$ and interpolation function, *g* are given as

2 $$\begin{aligned} \Omega =\frac{1}{2}\phi ^2(1-\phi )^2,\, g=3\phi ^2-2\phi ^3, \phi \in [0,1] \end{aligned}$$

The anisotropy, *A* is a function of Cartesian components of $\nabla \phi$ and is discussed in section “Phase field modelling of faceted anisotropy”.

For chemical potential the equation used is given by

3 $$\begin{aligned} {\dot{\mu }}=a\nabla \cdot D\nabla \mu -a\,\Delta c\,g'(\phi ){\dot{\phi }} \end{aligned}$$

where $D\equiv \phi D_L+(1-\phi )D_S,\, \Delta c\equiv c_L-c_S$ with coupling constant

4 $$\begin{aligned} \lambda \equiv \frac{3R_c\Delta c^2}{\delta } \end{aligned}$$

The constant parameter, *a*, is given in Table 1 and arises via a Legendre transformation to the grand potential energy formulation, equivalent to the Kim, Kim, Suzuki model,^26,27^ with bulk free energy given by

5 $$\begin{aligned} f_i(c)=\frac{1}{2}a(c-c_i)^2+\mu _0 c,\, i=S,L. \end{aligned}$$

Note that the model here is quadratic and that we avoid more detailed forms of free energy curves using, for example, Redlich-Kister expressions^28^, so that we may focus on the mechanism for crystal morphology formation. This is reasonable because beginning with a full Redlich-Kister model one can often extract a common tangent for the two phases and approximate the two free energy curves at the common tangent points by two second order polynomials. This is essentially the method as advocated and generalised in^29^—see Sec. D of the supplementary material for detail of the transformation between chemical potential and solute formulations.

The initial condition for the phase, $\phi$, at time $t=0$ is given in terms of the Cartesian coordinates, *x*, *y*, *z* as

6 $$\begin{aligned} \phi _{t=0}=\frac{1}{1+\exp \left[ -(\sqrt{x^2+y^2+z^2}-R_0)/\delta \right] } \end{aligned}$$

and for chemical potential

7 $$\begin{aligned} \mu _{t=0}=\mu _0-\phi a({\bar{c}} - c_S), \end{aligned}$$

where ${\bar{c}}=\alpha c_S+(1-\alpha )c_L$. This means that in the solid the chemical potential equals $\mu _0$, and at the far boundary the value of the chemical potential is $\mu _\infty =\mu _0-a({\bar{c}}-c_S)$ The constants used have the values given in Table 1

**Table 1:** Constant values for the phase field model.

Model parameters		
Variable	Description	Value
*M*	Characteristic diffusivity/mobility	1.0
$c_L$	Equilibrium liquid concentration	0.9
$c_S$	Equilibrium solid concentration	0.5
$\mu _0$	Equilibium chemical potential	1
*a*	Curvature of free energy	4
$D_L$	Liquid diffusivity range	[0.1, 10]
$D_S$	Solid diffusivity	$10^{-4}D_L$
$\lambda$	Coupling constant	$\Delta c^2$
$R_c$	Critical radius	10
$R_0$	Initial radius	20
$\delta$	Interfacial width	2
$\alpha$	Controls the boundary value for $\mu _\infty$	[0, 1]
$\epsilon$	Anisotropy stability factor	0.02

Before discussing the choice of anisotropy function we make the following observations about the model.

1. We have chosen to deploy a simple model so as to strip away as much complexity and so leave more clarity to see the basic mechanism of hopper crystal formation.
2. Using $\alpha \in [0,1]$ results in $\mu _\infty \in [1,-\,0.6]$, with the two values explored here: $\mu _\infty =0.2$ and $\mu _\infty =0.04$.
3. The constant critical radius is so named because an initial radius, $R_0<R_c$ in an isotropic model will theoretically melt—see^30^.
4. $\mu _0$ is the equilibrium chemical potential and an analogue of melting temperature in temperature driven solidification.
5. The parameter, *a*, originates in the coefficient of the quadratic term in the liquid and solid quadratic free energies as a function of solute concentration—see Sec. D of the supplementary materials.
6. $D_L,D_S$ are the diffusivity values for the liquid and solid respectively. On varying the mobility and identifying a revised characteristic diffusivity we found it more effective to (equivalently) vary these parameters and keep mobility, *M*, fixed at unity—see Sec. C of the supplementary material.
7. The coupling constant, $\lambda$, governing the relative surface and bulk free energy contributions.
8. The model does not include nucleation, so we introduce an initial small seed of radius, $R_0$.
9. The parameter $\delta$, known as the interface width lies at the heart of the phase field method and is so named because an equilibrium 1D simulation has a resulting interface width directly proportional to $\delta$. Under non equilibrium conditions though the analytical connection between $\delta$ and the resulting interface width is loosened.
10. It may be noticed that the chemical potential model presented here is practically indistinguishable from a thermal model for pure metal solidification. There is a key difference though: as well as there being no appreciable difference of thermal conductivity (diffusion) between a metal solid and liquid, the value of thermal conductivity in metals is much higher than solute diffusion (Lewis number typically $\approx 10,000$). This suggests that solidification of faceted cubic-type pure materials with analogously sufficiently small *heat* diffusivity, e.g. Bismuth, a semi-metal (wherein generally the melt is more metallic than the solid) may well exhibit similar solidification characteristics.

## Phase field modelling of faceted anisotropy

Surface energy is not a standard part of the thermodynamic description of the phases and it is necessary to find other means or rationale to construct this part of the free energy density. We follow the methods of Bollada et al^25^ that advocate the construction of the surface energy associated with arbitrary faceted crystal formation by using as a starting point the specification of the vertices (in 3D or 2D)—not the face normals. This is a departure from the standard way of forming faceted anisotropy functions, see for example^31^, and a later generalisation of this approach in^32^. The former is a 2D construction and precursor to^6^ and therefore also practically indistinguishable from^25^.

In Sec. A of the supplementary material we show the connection with (and differences from) the spherical harmonic expansion method discussed in^33^ in the context of non-faceted dentritic solidification.

In^25^ the authors advocate two approaches to numerical implementation of this function, which is discontinuous in the first derivative. One is to approximate derivatives of max function using a finite difference scheme. The other to approximate the max function (infinity norm) by a large, but finite valued, p-norm, which eliminates the issue occurring on a flat face. A separate issue for phase field is that of the sharp corners where the normal is not defined. Both problems may be tackled by the methods of^6^ or^25^, which approach the problem using a two pronged approach: namely, to make the faces (edges in 2D) curved; and make the vertices rounded. We find that for a special case of a cube one approach may be used, which allows a single parameter, $\epsilon$, to generate both rounded faces and corners.

The model we adopt for inclusion in Eq. (1) is given by

8 $$\begin{aligned} A=\sum _i\sqrt{X_i^2+\epsilon ^2 \left( X_1^2+X_2^2+X_3^2 \right) }, \end{aligned}$$

where $X_i\equiv \frac{\partial {\phi }}{\partial {x^i}},i=1 \ldots 3$. The parameter, $\epsilon$ (see Table 1, has the effect of regularising (smoothing) the corners of the cube and allowing the equilibrium cube surfaces and edges to be convex. Setting $\epsilon =0$ formally sets the equilibrium anisotropy to a perfect cube with consequent numerical instability. That is:

9 $$\begin{aligned} A=|X_1|+|X_3|+|X_3|=\max \left( \{\textbf{p}_i\cdot \nabla \phi ,i=1\ldots 8\}\right) \end{aligned}$$

where $\textbf{p}_i,i=1 \ldots 8$ are the vertices of a cube: $(\pm 1,\pm 1,\pm 1)$.

## Numerical considerations

We adopt the numerical approach of^34^ and all but one of the simulations use a mesh size and interface width parameter, $\Delta x=\delta =2$. Adaptive time stepping, $\Delta t$, is adjusted to be as large as possible without affecting the stability and was typically in the range [0.001, 0.01] for the larger step size, $\Delta x = 2$. The number of time steps in a simulation are typically $< 10^5$ giving a dimensionless simulation time $t<1000$. To save computation time we use an eighth domain with symmetry condition at the boundaries through the origin and also at the far boundary, set large enough to not compromise the chemical potential field (at least twice the size of the crystal surface).

## Results

### Hopper crystals

**Figure 1:**
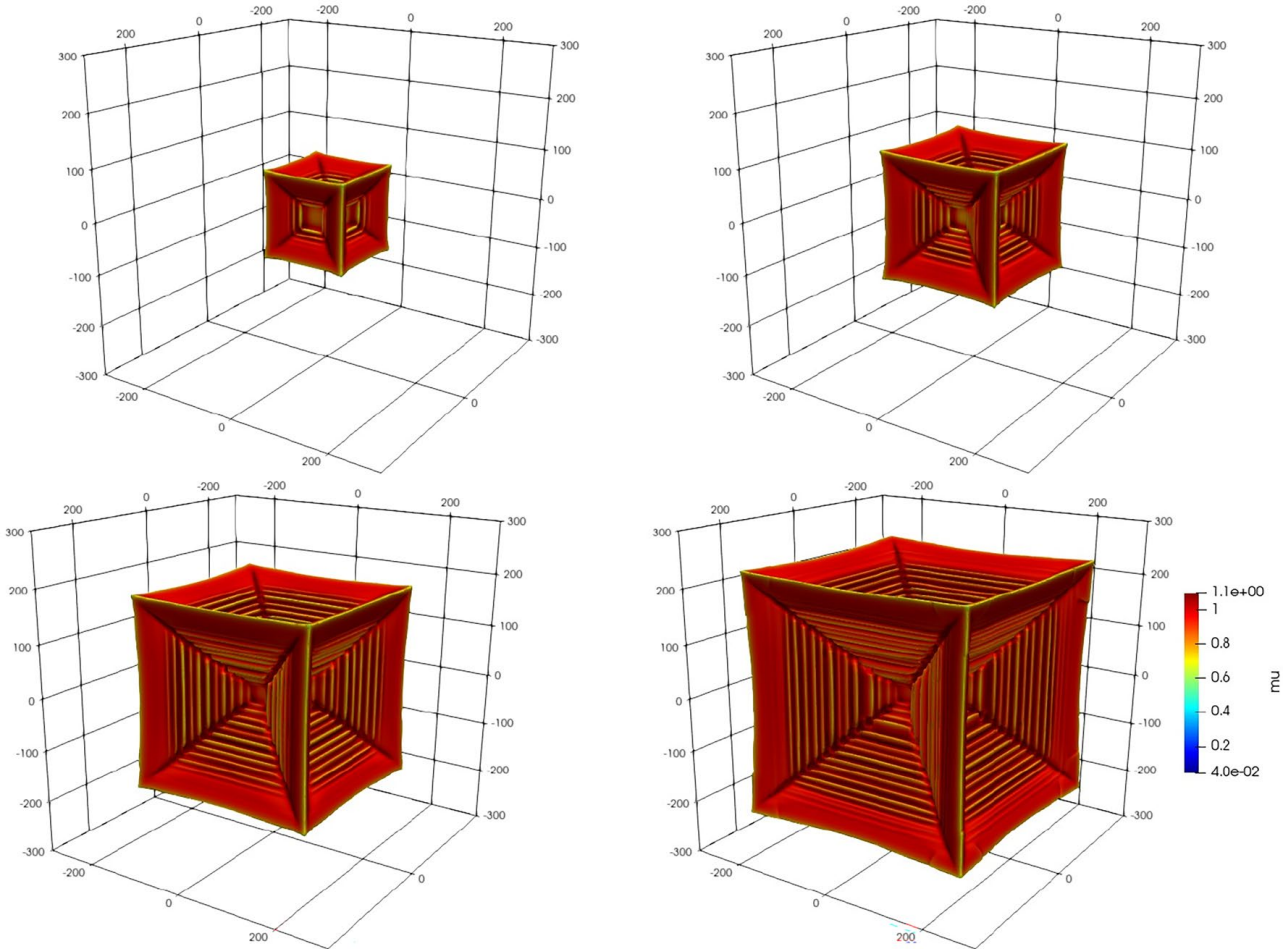
Growth of hopper crystal from seed in time intervals proportional to powers of 2 for parameters $D_L=1/12,\mu _\infty =0.04$.

As discussed in Sec. C of the supplementary material, in this non dimensional model, a reduction in diffusivity is equivalent to an increase in mobility: the response to a given driving force. The balance between the rate of growth and the rate of chemical diffusion reaches a critical point when a hollow forms in a flat face inhibiting diffusion and allowing build up of solute. Moving from the top of the left hand column Fig. 1 shows the growth from seed towards a mature hopper crystal, where the characteristic stepping is clearly shown. The parameters used in Fig. 1 are $\mu _\infty =0.04,D_L=1/12$.

**Figure 2:**
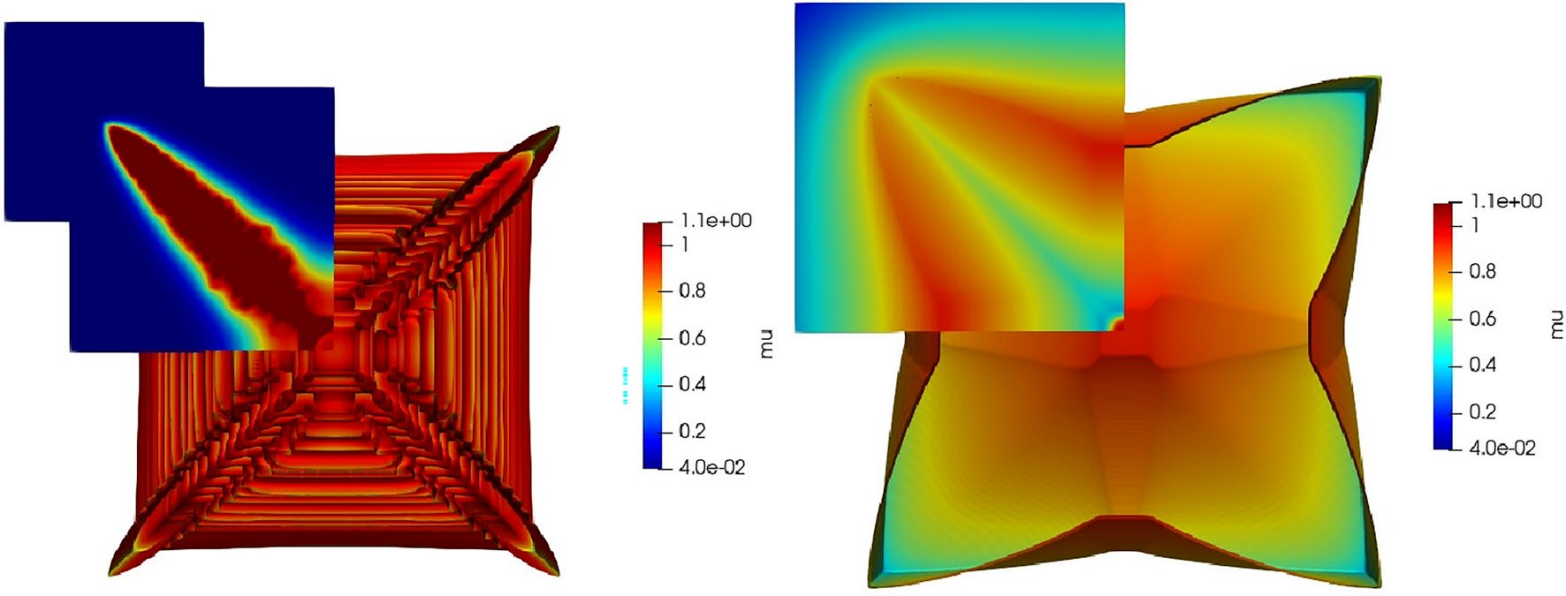
For chemical potential fixed at $\mu _\infty =0.04$ (at the final time step from Fig. 1), the results compare diffusivity, $D_L=1/12$ (left), with $D_L=4$ (right) along a projection normal to a Cartesian axis and through the origin (together with the surface of the crystal). This results supports the claim that the narrow boundary layer around the hopper crystal, being much smaller for lower diffusivity, is critical for this morphology.

In Fig. 2 we again fix the chemical potential at the boundary, to be $\mu _\infty =0.04$, but vary the diffusivity—both $D_L=1/12$ and $D_L=4$. The effect of the higher diffusivity of chemical potential is that the hopper crystal becomes more dendritic in morphology. But note that this is a $<111>$ dendrite (i.e. 8-pronged, growing towards the corners of the cube) rather than the more conventional $<100>$ dendrite (i.e. 6-pronged, growing towards the faces of the cube). These figures included a cross sectional plane so as to examine chemical potential away from the surface. Here we see the low diffusivity in the hopper crystal traps the layer close to the surface whereas the high diffusivity extends substantially far from the solid surface (n.b. these plots are in perspective and as such falsely appear to make the cross section dendrite extend further than the cube edge).

In Fig. 4 we reproduced the hopper $D_L=1/12,\mu _\infty =0.04$ (depicted in Fig. 1 at $\Delta x=2$) at a twice higher resolution mesh, $\Delta x=1$, to examine the possibility of numerical artefacts or contributions to the results. The hopper morphology was unaffected by this, confirming the mesh size was sufficient for examining the hopper morphology. The surface value for $\mu$ is close to the equilibrium value $\mu _0=1$, as expected, but inside the dendrite larger values of $\mu >1$ are achieved.

**Figure 3:**
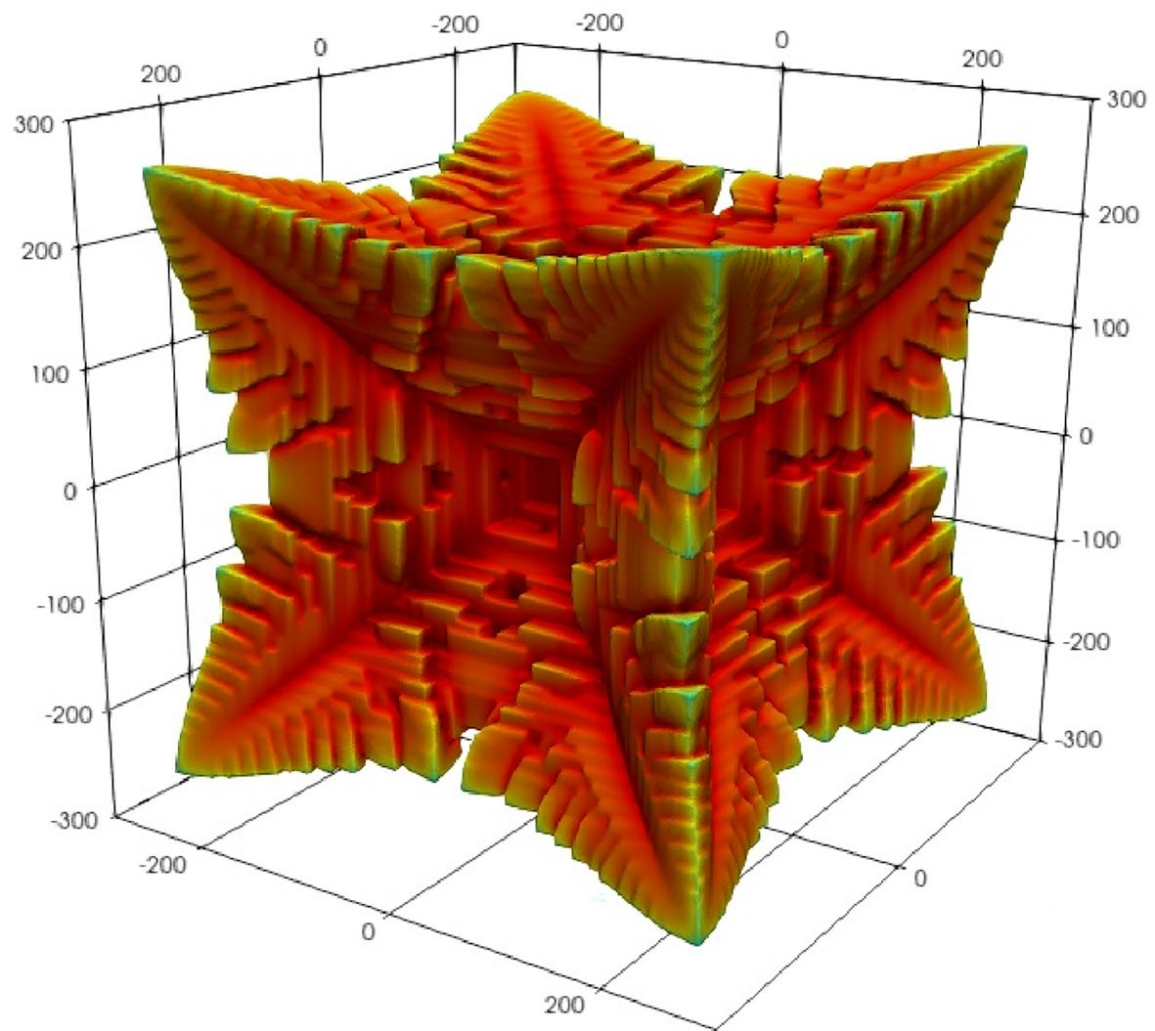
A fractal looking 8-vertex dendrite is produced for a diffusivity, $D_L=1/2$, intermediate between the cases illustrated in Fig. 2 ($D_L=1/12$ and $D_L=4$).

**Figure 4:**
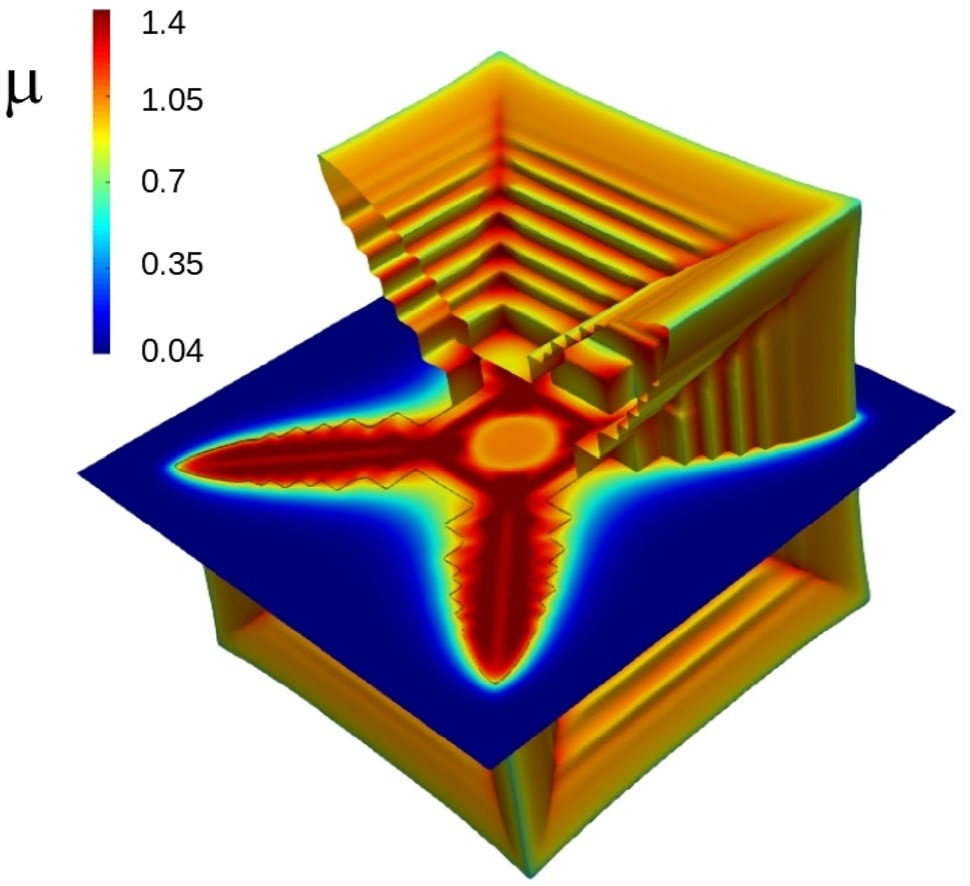
A cut out of the hopper crystal $D_L=1/12,\mu _\infty =0.04$ showing the tight boundary layer around the cross sectional dendrite and also the stepping (both in the surface and the black contour line at $\phi =\frac{1}{2}$ in the cross section).

Here we note that the fractal appearance of Fig. 3 is associated with a relative diffusivity (see Sec. C of the supplementary material for further discussion of our use of the term “relative diffusivity”) halfway between solute diffusion and phase mobility (see Sec. C in the supplementary material) where the interplay between solute and phase change is more complex. Figures 3 and 1 also serve to illustrate the scale of the simulation from, in this case, a nucleus of radius 20 to a size about ten times larger. As noted, the crystal image in Fig. 15b of^13^, presents a hopper crystal in salt, which, we may assume, has a narrow boundary layer of depleted salt solution formed around the hopper crystal face inhibiting growth. In alloys, a similar mechanism is that the face advances faster than the solute can redistribute and consequentially inhibits the growth at the centre of the face, which it turn restricts growth further.

Though being a reasonable explanation for departure from a flat face, this does not fully explain the formation of a hopper morphology, which appears to maintain the integrity of the edges of the cube. One might expect that as the driving forces increase, then the edge too will be unable to reject excess solute. This does not appear to be the case: in Fig. 2, we see that as mobility *decreases* the morphology changes from hopper to a smooth 8-cornered dendritic. A case, intermediate between these two, is the highly fractal 8-cornered dendrite shown in Fig. 3, and bears some similarity with the images found in^8^.

In these simulations (e.g. Fig. 2) we see, by inspection of the colour bar, that despite the chemical potential playing a role virtually identical to that of temperature^27^, the solid region existing above the equilibrium chemical potential, $\mu >\mu _0=1$. The likely reason for this is the much lower chemical diffusivity seen here compared to thermal conductivity in metal solidification, combined with the hopper morphology trapping the chemical potential within narrow regions. For evidence of this within our simulations, see the region in the cross sectional area of left hand plot of Fig. 2 which has $\mu >\mu _0$, but which is not present in the ($48 \times$) higher diffusivity plot on the right of Fig. 2.

**Figure 5:**
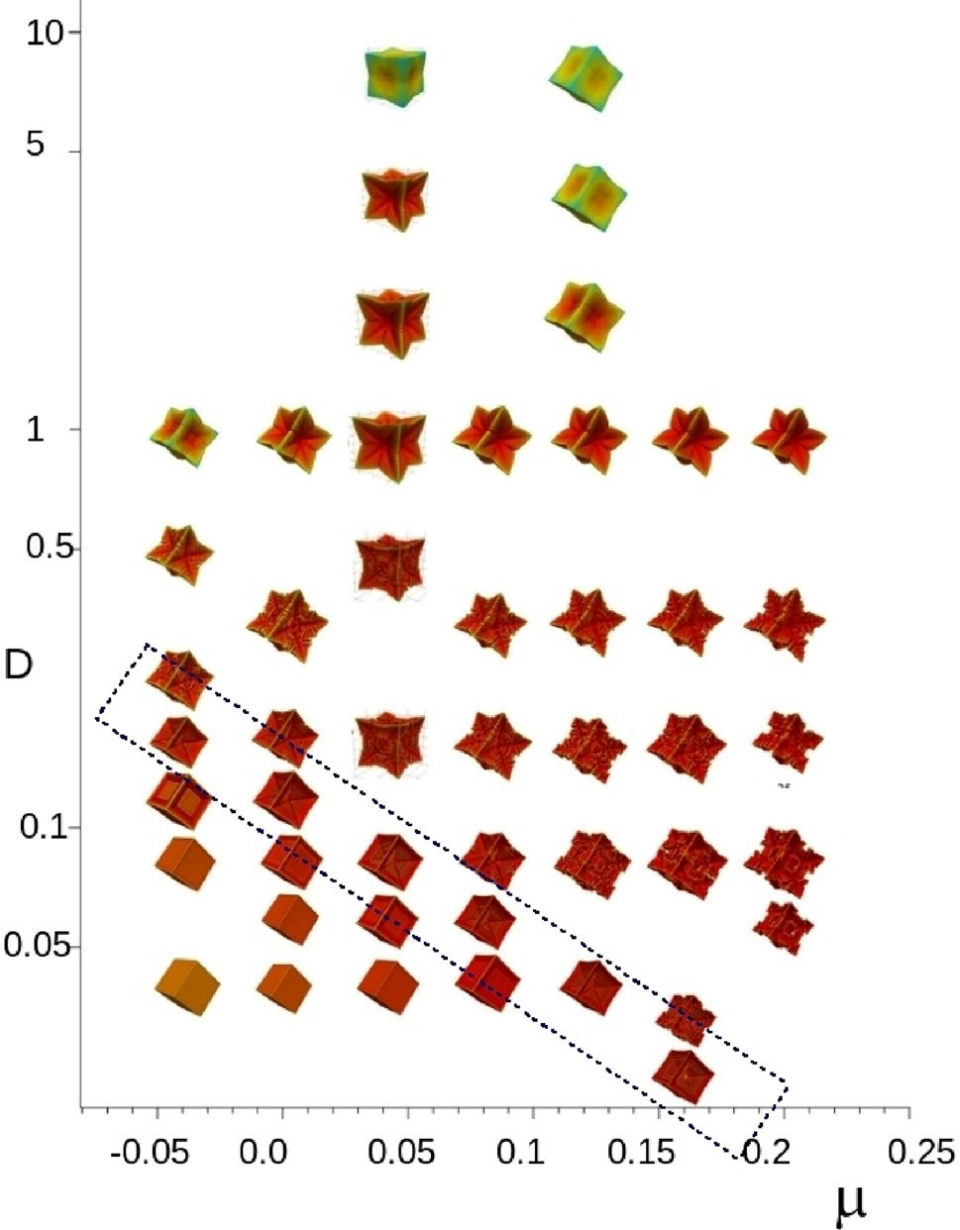
Morphology plot for a two parameter range of both $\mu _\infty \in [-\,0.05,0.2]$ and $D_L\in [0.02,16]$. There is a line of hopper crystals in the lower left of the diagram enclosed by a dashed box. Moving to the left creates a larger driving force; moving vertically down represents an increased mobility.

### Other (intermediate) morphologies

A key result of this paper is that cubic hopper crystals lie at the other extreme from the equilibrium cube and yet there is a plethora of other morphologies lying between the two extremes (please see also the video supplied in the supplementary materials). In the previous subsection we fixed the boundary value for chemical potential at $\mu _\infty =0.04$ (and all other parameters) and varied only the relative diffusivity between solute and phase field. One difficulty we experienced when straying too far from this series of results with fixed $\mu _\infty$ is that the resulting interface begins to vary with subsequent unstable results either caused by too small an interface width or, indeed, too large.

By extending our simulations to parameters beyond those depicted in Fig. 2 to those shown in Fig. 5, we see a narrow band where hopper growth appears. This suggests that it will be difficult, if not impossible, to grow in most pure metal alloys, even with underlying faceted morphology (at the very least one requires chemical diffusion, which of course is absent in a pure metal as there is no chemical species to diffuse to balance the innate mobility of the alloy). The hopper images observed in^8^ confirm that metal alloy solidification can also produce hopper crystals. Non-faceted solid-solution alloys cannot form hopper crystals since faceted anisotropy near equilibrium is a prerequisite for hopper formation. The question remaining is whether all alloys which can form (e.g. cubic) faceted morphology (e.g. intermetallic) can also form hopper-like structures. In a simulation we have the luxury of adjusting the diffusivity (formally identical to adjusting the mobility in this model), but in physical alloys we may only control temperature gradients and alloy concentration.

We now address the effect of changing the interface width on the resulting morphology. The results are not invariant under change of input interface width, $\delta$ though qualitatively the results are the same but translated. For example a hopper crystal can be found with $\delta =1.8$ ($90\%$ the value for the simulation in Fig. 1) at $D_L=0.07,\mu _\infty =-0.024$. On the other hand, consider again the parameters that return a hopper crystal: $\mu _\infty =0.04; D_L=1/12; \delta =2,R_c=10,R_0=20$. We find that the following parameters also return a hopper crystal: $\mu _\infty =0.04; D_L=1/12; \delta =1,R_c=5,R_0=10$. That is to say, a change of $\delta$, when interpreted as a mere change of length and time scale, leaves the equations formally unchanged—see Sec. E of the supplementary material for further discussion.

Once we have found a single hopper crystal we extended the result to a series of hopper crystals located along the line extending from $(D_L=0.2,\mu _\infty =-0.05)$ to $(D_L=0.04,\mu _\infty =0.12)$. The existence of such a relation is also suggested by a rescaling argument whereby a given pair of values, $D_L$ and $\mu _\infty$ can be transformed to another pair by choosing a new diffusion scale and interface width, $\delta$.

### Observations on the variety of morphologies

We have included as supplementary material a single video which sequentially explores part of Fig. 5. Initially, the diffusivity is large ($D_L=20$) and so the chemical potential field spreads out beyond the crystal surface and the resulting morphology is a near equilibrium cube. As the diffusivity is reduced, the $\mu$-field develops a tighter boundary layer eventually becoming comparable in width to the phase field interface width itself. The limiting case is the hopper figure itself, beyond which the crystal begins to break up (not shown). Intermediate between the equilibrium cube and hopper crystal are dendritic-like structures with an increasing fractal like surface. The animation then explores the lateral direction by incrementally bringing the boundary chemical potential, $\mu _\infty$ towards $\mu _0=1$ via the parameter $\alpha =0.4$ to 0.7 (using $\mu _\infty =(8\alpha -3)/5$ so that $\mu _\infty \in [0.04,0.54]$). The result morphologies are quite distinct from the morphologies produced by changing diffusivity alone. The mechanism behind the rich variety of morphologies is not easy to classify quantitatively. Yet, together with the observation that the equilibrium morphology (cube in this case) is achieved via either high enough diffusivity *or* small driving force due to small enough difference $\Delta \mu \equiv |\mu _0-\mu =\mu _\infty |$, the other extreme is the hopper morphology beyond which the simulation is unstable

## Octahedral “hopper” shapes

**Figure 6:**
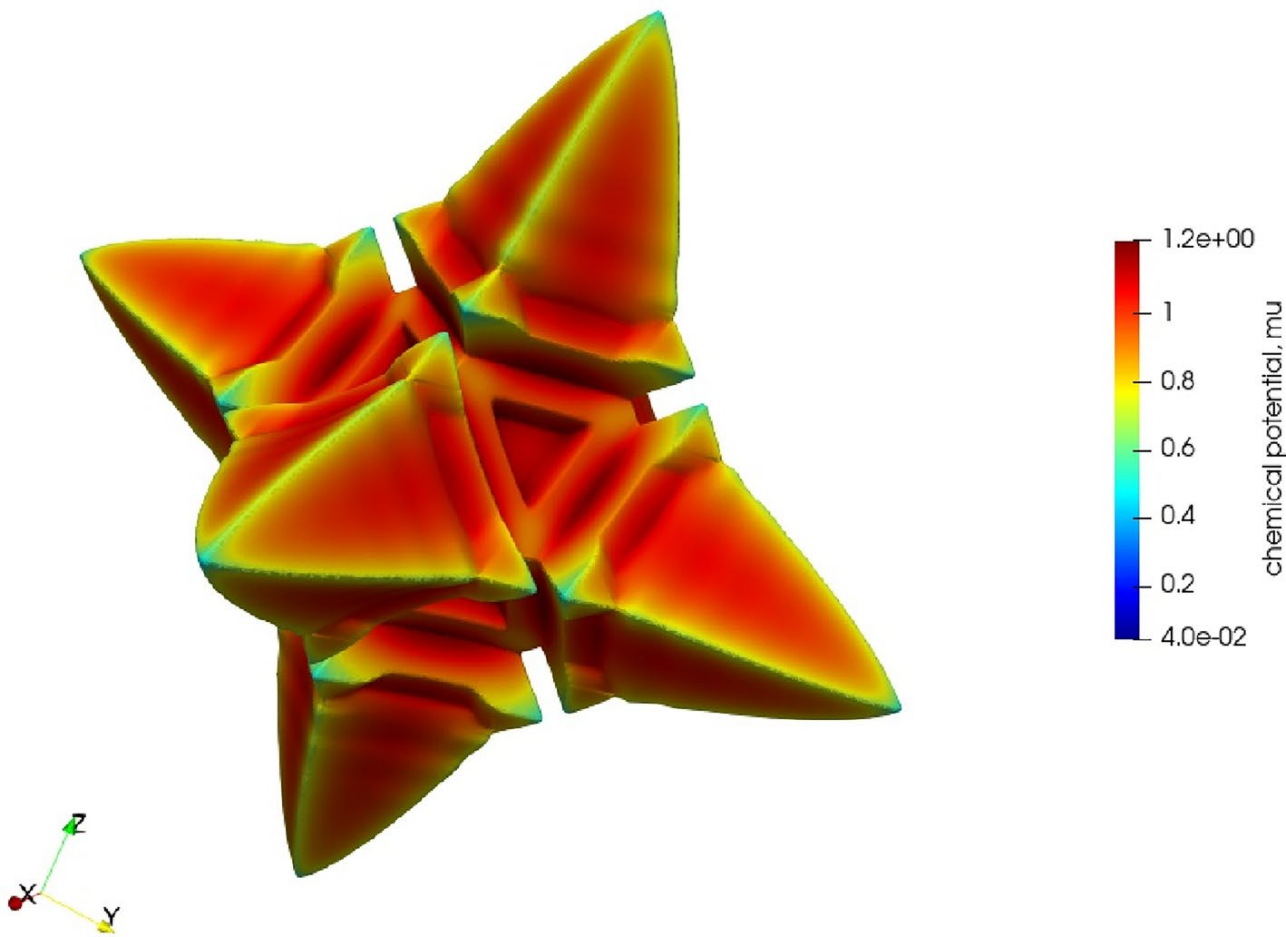
Octahedral growth using the same bulk parameters as for cubic growth.

The foregoing study of out of equilibrium driven cubic morphology poses the question of other primitive crystal structures. On application of the same thermodynamic model that returns the hopper cube ($\mu _\infty =0.04,d_L=1/12$ to an octahedral anisotropy (perfect octahedron under near equilibrium conditions) we find the result depicted in Fig. 6. An octahedron can be seen as the opposite of cubic growth where the strong growth directions of the cube are the weak directions of octahedron, and vice versa.

Such morphology has been observed in figure 1h of^35^ by SEM at a scale of about 1$\mu$m for each crystal. In both^35^ and Fig. 6 we observe that the inward pointing faces must necessarily align (closely) with an allowed facet direction and, as such, appears to create an inverted triangle in the hollow. Thus, suggests that in the cubic hopper growth, the apparent alignment of the hollow to its adjacent outer edge is due to the symmetry in the cubic faces and that growth must remain (at least approximately) in the allowed outward normal directions only.

## Summary and comment

The central result of this paper is the final frame of the series of simulation results in Fig. 1, but the primary message is that we have established that hopper morphology can arise theoretically in faceted materials for alloys that can form facets. The other major player in the model is kinetic mobility, as there is an intimate relation between the effective diffusivity of chemical potential and the kinetic mobility. In our model we only found a limited combination of parameters, within the set we chose, to allow hopper growth. It may still be the case that, given a particular intermetallic alloy capable of faceting, it will not be able to produce a hopper crystal since, in that case, we will only have a boundary solute condition and undercooling to vary. Thus, it remains a subject of further research to examine real materials together with necessarily estimates for the kinetic mobility.

### Supplementary Information

Supplementary Information 1.

Supplementary Information 2.

Supplementary Legends.

## Data Availability

Reproduction of the results in this paper can be obtained from our code at https://github.com/prepcb/PhaseField. Improvements to the code for general public use are ongoing and are projected to be complete by the end of 2023.

## References

[1] Peng D, Osher S, Merriman B, Zhao H-K (1970). The geometry of Wulff crystal shapes and its relations with Riemann problems. Contemp. Math..

[2] Taylor J, Cahn J (1998). Diffuse interfaces with sharp corners and facets: Phase field models with strongly anisotropic surfaces. Phys. D.

[3] Sekerka RF (2005). Equilibrium and growth shapes of crystals: How do they differ and why should we care?. Cryst. Res. Technol..

[4] Albani M, Bergamaschini R, Salvalaglio M, Voigt A, Miglio L, Montalenti F (2019). Competition between kinetics and thermodynamics during the growth of faceted crystal by phase field modeling. Phys. Status Solidi (b).

[5] Miura H (2013). Anisotropy function of kinetic coefficient for phase-field simulations: Reproduction of kinetic wulff shape with arbitrary face angles. J. Cryst. Growth.

[6] Debierre J-M, Karma A, Celestini F, Guerin R (2003). Phase-field approach for faceted solidification. Phys. Rev. E.

[7] Bollada P, Jimack P, Mullis A (2018). Faceted and dendritic morphology change in alloy solidification. Comput. Mater. Sci..

[8] Xian J, Belyakov S, Britton T, Gourlay C (2015). Heterogeneous nucleation of Cu6Sn5 in Sn-Cu-Al solders. J. Alloy. Compd..

[9] Morizane K, Witt AF, Gatos HC (1966). Impurity distributions in single crystals: I. Impurity striations in nonrotated crystals. J. Electrochem. Soc..

[10] Jackson K, Uhlmann D, Hunt J (1967). On the nature of crystal growth from the melt. J. Cryst. Growth.

[11] Yao WR, Lu WHA, Hogan L (1999). Growth morphology of primary silicon in castal? Si alloys and the mechanism of concentric growth. J. Cryst. Growth.

[12] Desarnaud J, Derluyn H, Carmeliet J, Bonn D, Shahidzadeh N (2018). Hopper growth of salt crystals. J. Phys. Chem. Lett..

[13] Stefanescu D, Alonso G, Gonzalez R, Suarez R (2020). Hopper-skeletal and hemispherical crystallization of graphite in iron-carbon-silicon alloys. Carbon.

[14] Amelinckx S (1953). Xxxvii. A dislocation mechanism for the growth of hopper crystal faces and the growth of salol crystals from solution and from the melt. Lond. Edinb. Dubl. Philos. Mag. J. Sci..

[15] Han P, Payne D (1990). Crystal growth of high tc superconductors in the system Bi-Ca-Sr-Cu-O. J. Cryst. Growth.

[16] Dickinson SR, McGrath KM (2003). Switching between kinetic and thermodynamic control: Calcium carbonate growth in the presence of a simple alcohol. J. Mater. Chem..

[17] Zhu J, Duan W, Sheng Y (2009). Uniform pbs hopper (skeletal) crystals grown by a solution approach. J. Cryst. Growth.

[18] Zhang J, Zhang S, Wang Z, Zhang Z, Wang S, Wang S (2011). Hopper-like single crystals of sodium chloride grown at the interface of metastable water droplets. Angew. Chem. Int. Ed..

[19] Pettit D, Fontana P (2019). Comparison of sodium chloride hopper cubes grown under microgravity and terrestrial conditions. NPJ Microgravity.

[20] Yin H, Wang Q, Chen G (2014). Probing the growth mechanism of pbte hopper-like crystal and ultra-long nanowires with rough surface synthesized through acetone-assisted solvothermal method. Chem. Eng. J..

[21] Hu Y, Xu C, Zhang H, Wang Y, Deng J, Zhang H (2017). Catalytic performance of ferrierite with different crystal grain sizes during skeletal isomerization of 1-butene. Chem. Sel..

[22] Zhao S, Guo T, Chu Z, Li Y, Xu W, Ran G (2021). Growth of hopper-shaped cspbcl3 crystals and their exciton polariton emission. RSC Adv..

[23] Ge X, Gu CD, Lu Y, Wang XL, Tu JP (2013). A versatile protocol for the ionothermal synthesis of nanostructured nickel compounds as energy storage materials from a choline chloride-based ionic liquid. J. Mater. Chem. A.

[24] Huang D, Liu H, Li T, Niu Q (2019). Template-free synthesis of nio skeleton crystal octahedron and effect of surface depression on electrochemical performance. J. Sol-Gel. Sci. Technol..

[25] Bollada PC, Jimack PK, Mullis AM (2021). A vertex based approach to crystal facet modelling in phase field. Comput. Mater. Sci..

[26] Kim SG, Kim WT, Suzuki T (1999). Phase-field model for binary alloys. Phys. Rev. E.

[27] Plapp M (2011). Unified derivation of phase-field models for alloy solidification from a grand-potential functional. Phys. Rev. E.

[28] Moelans N, Blanpain B, Wollants P (2008). An introduction to phase-field modeling of microstructure evolution. Calphad-Comput. Coupl. Phase Diagr. Thermochem..

[29] Choudhury A, Kellner M, Nestler B (2015). A method for coupling the phase-field model on a grand-potential formalism to thermodynamic databases. Curr. Opin. Solid State Mater. Sci..

[30] Wu W, Montiel D, Guyer J, Voorhees P, Warren J, Wheeler D, Granasy L, Pusztai T, Heinonen O (2021). Phase field benchmark problems for nucleation. Comput. Mater. Sci..

[31] Amar MB, Pomeau Y (1988). Growth of faceted needle crystals: Theory. Europhys. Lett. (EPL).

[32] Boukellal AK, Elvalli AK Sidi, Debierre J-M (2019). Equilibrium and growth facetted shapes in isothermal solidification of silicon: 3d phase-field simulations. J. Cryst. Growth.

[33] Dantzig JA, Napoli PD, Friedli J, Rappaz M (2013). Dendritic growth morphologies in Al-Zn alloys? Part ii: Phase-field computations. Metall. Mater. Trans. A.

[34] Bollada PC, Goodyer CE, Jimack PK, Mullis AM, Yang FW (2015). Three dimensional thermal-solute phase field simulation of binary alloy solidification. J. Comp. Phys..

[35] Ho J-Y, Huang MH (2009). Synthesis of submicrometer-sized Cu2O crystals with morphological evolution from cubic to hexapod structures and their comparative photocatalytic activity. J. Phys. Chem. C.

