# Supplementary material for: Phase field modelling of hopper crystal growth in alloys
Source: Sci Rep. 2023 Aug 3;13:12637. doi: 10.1038/s41598-023-38741-2 (PMC10400641; doi:10.1038/s41598-023-38741-2)
Supplement: Supplementary file 2 — Supplementary Information 2. [file 41598_2023_38741_MOESM2_ESM.pdf]

A Connection with the standard approach to anisotropy

In this supplementary text we relate our approach to formulating faceted anisotropy with another natural extension to the standard form of anisotropy found in [1].

A generalisation of the 4-fold 2D anisotropy, $A = 1 + \epsilon \cos 4\theta$ (which is well known to lead to dendritic structures in 2D), can be made by considering A to be just two terms in a more general Fourier series expansion

$$A = a_0 + \sum_{i=1}^{n} a_i \cos 4i(\theta - \theta_0), \qquad (1)$$

where arbitrary orientation is included by using a phase angle, θ_0. Clearly, $A = 1 + \epsilon \cos 4\theta$ is a particular case of Eq. 1 (with $a_0 = 1, a_1 = \epsilon, \theta_0 = 0$ and $a_i = 0$ for $i > 1$). It may be observed that changing the sign of ϵ changes the orientation of the dentrite. This motivated [1] to consider a spherical harmonic based formulation in 3D that encompasses a variety of cubic type crystals, in particular all of: $< 100 >, < 110 >$ and $< 111 >$.

For our purposes, we can ask whether the scheme of harmonic expansion, necessarily continuous in all its derivatives, provides a solution to the discontinuity encountered in facet anisotropy. By studying the 2D case of square anisotropy, we find that this is not the case.

For a perfect square, we start with the anisotropy

$$A = |\cos\theta| + |\sin\theta| \qquad (2)$$

and find the Fourier approximation

$$A = a_0 + \sum_{i=1}^{n} a_i \cos(4i\theta) \qquad (3)$$

where $a_0 = 4/\pi$ and

$$a_i = -\frac{8}{16i^2 - 1}, i = 1..n \qquad (4)$$

Fig. 1 shows the effect of taking a five term Fourier expansion on the anisotropy for a square. Although the anisotropy, A, seems satisfactorily captured (to the eye), the first derivative visually differs, resulting in a Wulf plot with cusps about the corners (see [2]). Such cusps signal instability for numerical computations.

The approach taken in this paper approximates Eq. 2 anisotropy by writing

$$A \approx \sqrt{\sin^2\theta + \epsilon^2} + \sqrt{\cos^2\theta + \epsilon^2} \qquad (5)$$

We can compare the two types of approximations to the true anisotropy given by Eq. 2, by examining the two Figs 1 and 2. Both approximation methods give a visibly different Wulf shape to the perfect square, but the approximation, Eq. 5, is still a legitimate anisotropy leading to a legitimate Wulf shape. On the other hand, the resulting Wulf shape following from Eq. 3 gives a Wulf shape with an ambiguous function of θ. Such functions need truncating to keep the shape convex [3].

Thus, we conclude that Fourier series, and by extension, spherical harmonic expansion does not naturally lend itself to facet modelling.

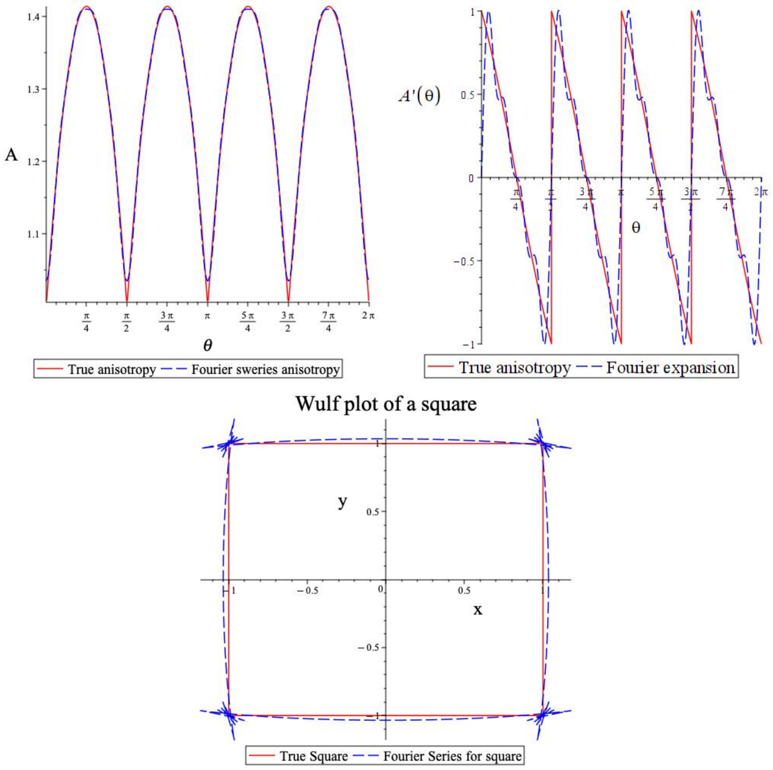

Figure 1: Top left we superimpose the true anisotropy for the square given by Eq. 2 (in red), with a 5 term Fourier expansion (dashed blue). Top right: the respective first derivatives of the anisotropy, resulting in (below) the respective Wulf plot

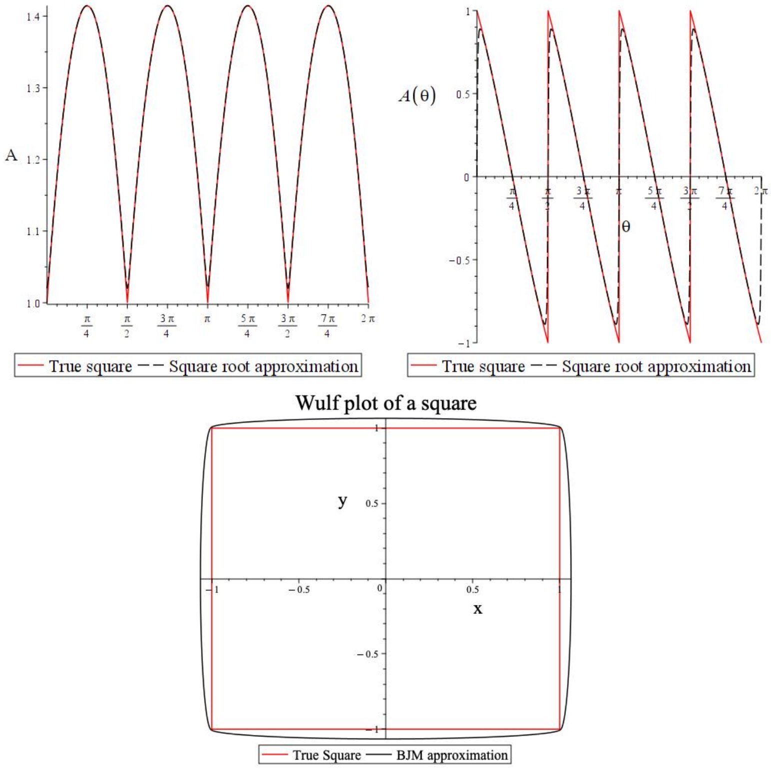

Figure 2: We superimpose the approximated Wulf plot using Eq. 5 with $\epsilon = 0.02$ on the true square.

B From non-dimensional equations to dimensional

Writing the phase equation as

$$\dot{\phi} = -M \frac{\delta F}{\delta \phi} \tag{6}$$

for some dimensional mobility parameter, M, where the grand potential energy is given by

$$F = \int_V \left[W \left(\frac{1}{2} \delta^2 A^2 + \Omega \right) + \omega_B \right] \mathrm{d}x \, \mathrm{d}y \, \mathrm{d}z \tag{7}$$

implies the evolution for phase

$$\dot{\phi} = MW\delta^2 \nabla \cdot \frac{\partial}{\partial \nabla \phi} \left(\frac{1}{2} A^2 \right) - MW\Omega'(\phi) - M\omega_B'(\phi). \tag{8}$$

Here, W and ω_B both have units of energy per unit volume. Comparing the dimensional equation, Eq. 8, with the non-dimensional equation , Eq. 1 and Eq. 4 imply

$$MW\delta^2 = 1, \, M = \frac{1}{\lambda \delta^2 \Delta c} \equiv \frac{1}{3R_c \Delta c^2 \, \delta} \tag{9}$$

and so fixes $W = 3R_c\Delta c^2/\delta$.

Now $[\delta]$ has units of length; $[MW\delta^2] = m^2/s$ has units of diffusion; and $[W] = J/m^3$ has units of energy per volume (to match μ). So, implicit in the above is a choice of length scale, diffusion scale, and energy/volume scale associated with the physical problem. Label these: L^*, D^*, E^* respectively gives, for example:

$$\begin{aligned} \delta &= 2L^* \\ R_c &= 10L^* \\ MW\delta^2 &= D^* \\ W &= \frac{3R_c\Delta c^2}{\delta} E^* \end{aligned} \tag{10}$$

A typical value for these scales are given as

$$\begin{aligned} L^* &= 1 \times 10^{-9}\mathrm{m} \\ D^* &= 1 \times 10^{-9}\mathrm{m}^2/\mathrm{s} \\ E^* &= RT = 8.31 \times 1000K = 8310\mathrm{J/mol} \end{aligned} \tag{11}$$

The first two also imply a time scale of $T^* = 1 \times 10^{-9}s$. From the results we have simulated the growth from a seed of radius $20 \times L^*$ to mature size of about $400 \times L^*$ in a time of (see discussion of time stepping in Sec. 5) $t \sim 1000T^* = 1 \times 10^{-6}s$.

C Mobility and diffusion

In our model we state that an increase in mobility is equivalent to a decrease in solute diffusivity. Since the units of mobility and diffusivity or not the same this can not be true in general. However is true when the characteristic diffusivity associated with the phase change is proportional to mobility only.

To see this, consider the general phase field equation for isotropic growth

$$\begin{aligned}\frac{\partial \phi}{\partial t} &= -M\frac{\delta F}{\delta \phi} \\ &= MW\delta^2\nabla^2\phi + ME(\phi, c) \end{aligned} \tag{12}$$

where E includes all the non-gradient terms, then

$$D^* \equiv MW\delta^2 \tag{13}$$

has the units of diffusivity (m^2/s), W units of energy per volume, δ units of length. In the case W, δ constant then only M affects D^*. So that choosing a characteristic diffusivity, D^* equal to unity implies that changes in M are equivalent to rescaling solute diffusivity, D, in the solute equation:

$$\frac{\partial c}{\partial t} = \nabla \cdot D\frac{\delta F}{\delta c}. \tag{14}$$

D From Grand Potential to free energy formulation

In [4] the paper demonstrates the equivalence of the Grand Potential Energy formulation (GPE) with a free energy formulation (FE) with a number of examples including quadratic free energy functions, by Legendre transformations from FE to GPE. Here we do this in reverse and begin by defining the grand potential energy density (GPE) for the liquid and solid phase as a quadratic function of chemical potential, μ:

$$\omega_i = c_i(\mu_0 - \mu) - \frac{(\mu - \mu_0)^2}{2a}, \, i = L, S. \tag{15}$$

We now form the phase dependent bulk GPE by writing

$$\omega_B = g(\phi)\omega_L + (1 - g(\phi))\omega_S, \tag{16}$$

so that the contribution to the phase equation is

$$\omega'_B(\phi) = g'(\phi)(\mu_0 - \mu)\Delta c, \tag{17}$$

where there is cancellation of the quadratic term and we introduce the notation $\Delta c \equiv c_L - c_S$.

We intend to transform this equation to free energy form using the Legendre transformation

$$f_B = \omega_B + \mu c \tag{18}$$

where c is defined

$$\begin{aligned} c &= -\frac{\partial \omega_B}{\partial \mu} \\ &= -g\frac{\partial \omega_L}{\partial \mu} - (1-g)\frac{\partial \omega_S}{\partial \mu} \\ &= g(c_L + (\mu - \mu_0)/a) + (1-g)(c_S + (\mu - \mu_0)/a) \\ &= gc_L + (1-g)c_S + (\mu - \mu_0)/a. \end{aligned} \tag{19}$$

This implies that

$$\mu = a(c - [gc_L + (1-g)c_S]) + \mu_0 \tag{20}$$

and so we find on applying the transformation Eq. 18:

$$f_B(c) = \frac{1}{2}a(c - [gc_L + (1-g)c_S])^2 + c\mu_0 \tag{21}$$

with respective energies for each phase ($g = 0, 1$) implied to be

$$f_i(c) = \frac{1}{2}a(c - c_i)^2 + \mu_0 c, i = S, L. \tag{22}$$

The grand potential phase equation Eq. 1 may be written

$$\dot{\phi} = \nabla \cdot \frac{\partial}{\partial \nabla \phi}\left(\frac{1}{2}A^2\right) - \frac{\Omega'(\phi)}{\delta^2} - \frac{1}{\lambda \delta^2}\frac{\partial \omega_B}{\partial \phi} \tag{23}$$

and is transformed to the free energy phase equation

$$\dot{\phi} = \nabla \cdot \frac{\partial}{\partial \nabla \phi}\left(\frac{1}{2}A^2\right) - \frac{\Omega'(\phi)}{\delta^2} - \frac{1}{\lambda \delta^2}\frac{\partial f_B}{\partial \phi} \tag{24}$$

Despite their notational similarity, the partial derivative with respect to ϕ in Eq. 23 is with μ held constant, but in Eq. 24, the partial derivative with respect to ϕ is with c held constant.

A subtle point is that the form of f_B in Eq. 21, is necessarily different to the form of ω_B, defined in Eq. 16, to accommodate the fact that the two formulations are formally physically identical (that is, they are transformed from one variable space to another). In other words, the linear combination of GPEs for each phase in Eq. 16 does not correspond to a linear combination of free energy for each phase in Eq. 21.

We also have from Eq. 20

$$\dot{\mu} = a\dot{c} - a\Delta c g'(\phi)\dot{\phi} \tag{25}$$

But from Sec. 2 in the main text Eq. 3

$$\dot{\mu} = a\nabla \cdot D\nabla\mu - a\,\Delta c\, g'(\phi)\dot{\phi} \tag{26}$$

which implies the diffusion equation for solute, c

$$\dot{c} = \nabla \cdot D\nabla \frac{\partial f_B}{\partial c} \tag{27}$$

To complete the KKS model we define the bulk free energy

$$f_B = g(\phi)f_L(C_L) + (1 - g(\phi))f_S(C_S)) \tag{28}$$

For quadratic functions, f_L, f_C, the above equation is readily defined using

$$\mu = f'_L(C_L) = f'_S(C_S) \tag{29}$$

and the constraint

$$c = g(\phi)C_L + (1 - g(\phi))C_S \tag{30}$$

to give both fields C_L and C_S and hence $f_B(c, \phi)$ – see also [4, 5].

E Rescaled equations

Beginning with the unscaled phase equation, we introduce a new interface width and then proceed to rearrange and then scale away the changes and hence show that the scaled equations are formally the same as the unscaled equations. Thus

$$\frac{\partial \phi}{\partial t} = \nabla \cdot \frac{\partial}{\partial \nabla \phi}\left(\frac{1}{2}A^2\right) - \frac{\Omega'(\phi)}{\delta^2} - \frac{g'(\phi)(\mu_0 - \mu)\Delta c}{\lambda \delta^2} \tag{31}$$

with

$$\lambda \equiv \frac{3R_c \Delta c^2}{\delta} \tag{32}$$

becomes, after multiplication by $(\delta/\delta^*)^2$:

$$\begin{aligned}\left(\frac{\delta}{\delta^*}\right)^2 \frac{\partial \phi}{\partial t} &= \left(\frac{\delta}{\delta^*}\right)^2 \nabla \cdot \frac{\partial}{\partial \nabla \phi}\left(\frac{1}{2}A^2\right) \\ &\quad - \frac{\Omega'(\phi)}{\delta^{*2}} - \frac{g'(\phi)(\mu_0 - \mu)\Delta c}{\lambda \delta^{*2}}.\end{aligned} \tag{33}$$

Using a new length and time scale, with new differential operators,

$$\frac{\partial}{\partial t^*} = \left(\frac{\delta}{\delta^*}\right)^2 \frac{\partial \phi}{\partial t}, \ \nabla^* = \left(\frac{\delta}{\delta^*}\right)^2 \nabla \tag{34}$$

this can be written

$$\begin{aligned}\frac{\partial \phi}{\partial t^*} &= \nabla^* \cdot \frac{\partial}{\partial \nabla^* \phi}\left(\frac{1}{2}A^2\right) \\ &\quad - \frac{\Omega'(\phi)}{\delta^{*2}} - \frac{g'(\phi)(\mu_0 - \mu)\Delta c}{\lambda \delta^{*2}}\end{aligned} \tag{35}$$

with λ unchanged provided we choose the revise critical radius to be defined by

$$\frac{R_c^*}{\delta^*} = \frac{R_c}{\delta} \tag{36}$$

Finally, the new chemical potential equation is

$$\frac{\partial \mu}{\partial t^*} = a\nabla^* \cdot D^* \nabla^* \mu - a\, \Delta c\, g'(\phi) \frac{\partial \phi}{\partial t^*}. \tag{37}$$

Thus, if we use the same non-dimensional numerical value for δ in the unscaled Eq. 1 in the main text Sec. 2, as used for δ^* in the scaled equation, 31, and the numerical value for the constants in D in the main text Sec. 2 Eq. 3 are chosen precisely the same as in D^* in 37, then the implemented (non-dimensional) equations are identical.

References

[1] J. A. Dantzig, P. D. Napoli, J. Friedli, and M. Rappaz, "Dendritic growth morphologies in al-zn alloys?part ii: Phase-field computations," *Metallurgical and Materials Transactions A*, vol. 44, pp. 5532–5543, 2013.

[2] P. C. Bollada, P. K. Jimack, and A. M. Mullis, "A vertex based approach to crystal facet modelling in phase field," *Computational Materials Science*, vol. 192, p. 110331, 2021.

[3] R. F. Sekerka, "Equilibrium and growth shapes of crystals: how do they differ and why should we care?," *Crystal Research and Technology*, vol. 40, no. 4-5, pp. 291–306, 2005.

[4] M. Plapp, "Unified derivation of phase-field models for alloy solidification from a grand-potential functional," *Phys. Rev. E*, vol. 84, p. 31601, 2011.

[5] S. G. Kim, W. T. Kim, and T. Suzuki, "Phase-field model for binary alloys," *Phys. Rev E*, vol. 60(6), pp. 7186–7197, 1999.
