# Supplementary material for: Phase field modelling of hopper crystal growth in alloys
Source: Sci Rep. 2023 Aug 3;13:12637. doi: 10.1038/s41598-023-38741-2 (PMC10400641; doi:10.1038/s41598-023-38741-2)
Supplement: Supplementary file 3 — Supplementary Legends. [file 41598_2023_38741_MOESM3_ESM.pdf]

Video description

This video consists of 14 separate time dependent simulations, initially varying the diffusivity, $D_L = 20, 8, 4, 2, 1, 1/2, 1/4, 1/8, 1/12$ (from high to low) with the boundary condition fixed via $\alpha = 0.4$. The resulting morphology changes from near equilibrium cube towards a hopper passing through the intervening shapes. We then vary the boundary condition via the parameter, $\alpha = 0.45, 0.5, 0.55, 0.6, 0.7$, to reduce the thermodynamic driving force as μ_∞ tends towards μ_0.
